# Comparing the Effectiveness of Hands-on vs. Observational Training of Residents in Interlaminar Epidural Steroid Injections (ILESI) Using a High-Fidelity Spine Simulator

**DOI:** 10.7759/cureus.49829

**Published:** 2023-12-02

**Authors:** Geum Y Sim, Moorice Caparó, Giustino Varrassi, Christopher R Lu, Michael E Ding, Rohini Singh, Kateryna Slinchenkova, Naum Shaparin, Sarang S Koushik, Omar Viswanath, Andrew I Gitkind

**Affiliations:** 1 Physical Medicine and Rehabilitation, Albert Einstein College of Medicine, The Bronx, USA; 2 Pain Medicine, Paolo Procacci Foundation, Rome, ITA; 3 Pain Management, Albert Einstein College of Medicine, The Bronx, USA; 4 Anesthesiology, Albert Einstein College of Medicine, The Bronx, USA; 5 Anesthesiology, Creighton University School of Medicine, Phoenix, USA; 6 Anesthesiology, Valleywise Health Medical Center, Phoenix, USA; 7 Anesthesiology, Louisiana State University Health Sciences Center, Shreveport, USA

**Keywords:** epidural injections, residency, education, simulator, high fidelity

## Abstract

Introduction

The Accreditation Council for Graduate Medical Education (ACGME) requires that residents in the Physical Medicine and Rehabilitation (PM&R) residency observe or perform certain interventional procedures, one of which is an interlaminar epidural steroid injection (ILESI). While the traditional learning model relying heavily on observation is commonplace, it leaves the practice phase of learning to happen on real patients. High-fidelity simulation may be a worthwhile alternative as a training approach to increase physician comfort with the procedure and improve patient safety.

Methods

Current PM&R residents from two programs between their second and fourth year, inclusively, who lacked prior training experience in ILESI attended one hour of either: (1) an experimental arm of supervised hands-on training on a simulation device or (2) a control arm observing the procedures performed by an attending on the same device. Assignments were made based on resident schedule availability. Pre-training knowledge, training, and post-training knowledge were assessed at the Multidisciplinary Pain Clinic at Montefiore Medical Center. Participants were assessed on their procedural competence using an adapted version of a previously published grading checklist before the session. Participants also evaluated their confidence in performing the procedure prior to and after training. Data was analyzed using the Wilcoxon signed-rank test and the Wilcoxon rank-sum test. SAS Version 9.4 was used for analysis.

Results

Fifteen residents initially participated, but three residents dropped out at the 15-week follow-up. There was a significant increase in test scores in both arms immediately after the intervention (p=0.008 in control, p=0.016 in the experiment), with greater improvement shown in the hands-on training group (p=0.063). At the 15-week follow-up, there was no significant change in test scores in the control arm (p=0.969) while there was a decrease in the experiment arm (p<0.001).

Conclusion

Hands-on learning with high-fidelity simulation demonstrated more improvement for short-term motor-skill acquisition, while observational learning with repetition showed more benefits for long-term retention. Optimal procedural training should employ both educational modalities for best short- and long-term results.

## Introduction

Pain medicine is recognized as a multidisciplinary specialty and is a popular fellowship choice among Physical Medicine and Rehabilitation (PM&R) residents [1,2]. The Accreditation Council for Graduate Medical Education (ACGME) now requires PM&R residents to observe or perform certain interventional pain procedures [1]. The exposure to interventional pain medicine during PM&R residency allows for early acquisition of procedural skills, which may bridge learning gaps for trainees who pursue a fellowship in pain medicine. The traditional learning method for interventional pain procedures follows the paradigm of “see one, do one, teach one” and is based on the observation of appropriate visuo-motor behaviors of attending physicians [3]. Trainees then practice procedures on actual patients based on their vicarious learning. This method, however, may be falling out of favor, as it raises concern for patient safety when the initial slope of the learning curve takes place.

Compared with the traditional learning model, simulation-based medical education (SBME) has been shown to facilitate education of procedural skills among trainees in other fields [4]. High-fidelity simulation is used in healthcare to reproduce an actual patient scenario to a high level of realism [4]. SBME provides a safe environment for diligent practice, which results in improved skill retention, recall, and transferability to the real clinical environment [5]. While no interlaminar epidural steroid injection (ILESI)- specific studies analyzing the implementation of SBME have been published, applying SBME to training physicians for lumbar puncture showed increased confidence and reduced patient dose. No comments on information retention or demerits have been made [5].

Although there are many proposed stages involved in the teaching of procedural skills with SBME, a well-researched method is Peyton’s four-step approach, which consists of the following steps: demonstration, deconstruction, comprehension, and performance [6]. Steps 1 and 2 allow the trainee to observe the instructor perform the procedure in real time (demonstration) and receive detailed instructions while the instructor performs the procedure again (deconstruction). Steps 3 and 4 focus on the trainee’s ability to guide the instructor through the procedure (comprehension) and conduct the procedure independently (performance) [7]. In the setting of SBME, Peyton’s teaching approach allows for the reinforcement of learning, the opportunity for the instructor to provide feedback, and the trainee to correct errors [7]. A recent systematic review with meta-analysis evaluated the effectiveness of Peyton’s teaching approach compared to the traditional teaching approach and the primary finding was that Peyton’s approach was more effective on the acquisition of procedural skills at post-acquisition testing [6]. The incorporation of Peyton’s four-step approach with SBME can allow trainees to gain competency in procedural skills without harm to patients. 

Little is known regarding the ideal method of instruction for acquiring the skills and competency to perform interventional pain procedures during PM&R residency training. Studies have shown that simulation-based learning allows practice opportunities for residents while improving the safety and quality of patient care, posing no risk of errors or complications to the patient [3]. To our knowledge, the use of SBME in teaching PM&R residents interventional pain procedures has not yet been well established or investigated. Through the use of a high-fidelity spine simulator, residents can gain hands-on experience during the initial stage of procedural skill acquisition. Our study attempts to evaluate the comparative effectiveness of simulation training vs. traditional observational learning for teaching PM&R residents how to perform an ILESI, a commonly performed procedure for lumbar radiculopathy or lumbar stenosis. The ultimate goal is for trainees to demonstrate effectiveness and transfer procedural skills to the clinical environment while improving resident competency and comfort level. It is expected that those with more hands-on experience will perform better in the long and short-term evaluations. If the hypothesis is supported, future educational approaches can improve by producing the most competent providers and improving patient safety by allowing providers to practice on simulators first.

## Materials and methods

Study design

Due to the lack of studies evaluating ILESI education and knowledge retention, we conducted a nonrandomized comparison study of observational learning vs hands-on simulator training. All steps of the procedure for both arms were performed on the simulator, decreasing patient risk. The Institutional Review Board (IRB) at our hospital approved the study and granted IRB exemption (IRB Number: 2022-13816). This study was performed in accordance with the Helsinki Declaration of 1964 and its later amendments.

Setting and participants

Residents from two academic PM&R residency programs (Burke Rehabilitation Hospital, Montefiore Medical Center) were the subjects of the study. Inclusion criteria were as follows: 1) current resident status, 2) no prior experience in performing a lumbar ILESI, and 3) able to give informed consent. All participants received an explanation of the study procedures and written consent was obtained from all individuals prior to participation.

Intervention/educational modality

Residents participated in either a full hour of the experiment arm or the control arm led by the precepting faculty at Montefiore Medical Center Multidisciplinary Pain Clinic. The groups were not strictly assigned but were hosted as per resident availability on two different evenings. Two trained preceptors (dual board-certified in PM&R and Pain Medicine, current full-time faculty at an ACGME-accredited Pain Medicine fellowship program) were assigned to educate and grade the participants. Neither the preceptors nor participants were blinded due to the nature of the experiment. Peyton’s four-step approach was further modified to promote efficiency in learning. The experiment arm comprised hands-on simulation training in which the faculty performed a one-time demonstration of lumbar ILESI on a simulator while describing all necessary sub-steps (‘demonstration’ and ‘deconstruction’). Following faculty demonstration, participants in the experiment arm were allotted equal time to explain each sub-step while performing the procedure (‘comprehension’ and ‘performance’). The control arm consisted of observational training during which the faculty member demonstrated lumbar ILESI on a simulator three times while describing all necessary sub-steps. Participants in the control arm were not allotted any hands-on practice time. 

Simulator

**Figure 1:**
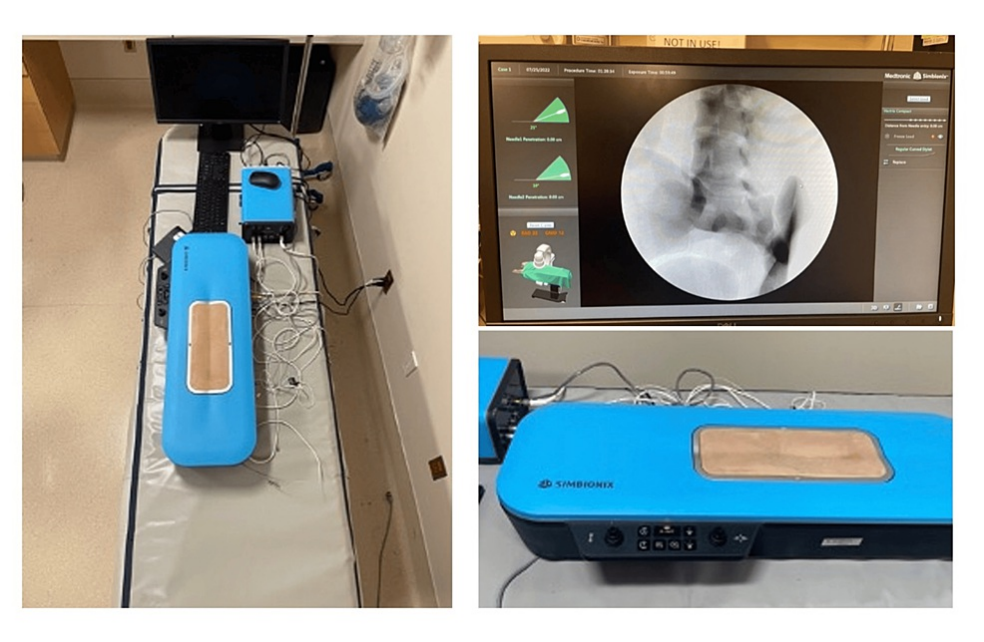
Simulator set up

Simulation training was conducted using a Simbionix Spine Mentor (product number SP013-02-18, produced by Medtronic) (Figure 1). Simbionix Spine Mentor (simulator) was provided by Medtronic, INC and returned upon study completion. All subjects used the same spine simulator consisting of a skin-mimicking rectangular surface with an accompanying joystick and simulator needle as well as an associated screen showing a C-arm view. Identification of spinal level views and orientation was performed with the joystick, with needle movements tracked on the screen settings allowed for either continuous imaging or for individual images taken using a footpad. The simulation needle came with accompanying syringes which could successfully simulate loss of resistance.

Scoring and outcome measurements

The subjects were given a pre-intervention and post-intervention questionnaire regarding their confidence levels and a one-on-one skills evaluation immediately before and after the educational modality (hands-on or observation), respectively. The skills evaluation was also administered again 15 weeks following the intervention to assess for retention. The questionnaire surveyed baseline trainee characteristics and self-perceived competence with the procedure, with subjects grading their perceived confidence on a five-point Likert scale ranging from “Not at all confident-0” to “Completely confident- 5”. The skills test consisted of performing an ILESI on the model with observation and grading by preceptors based on a checklist rubric adapted from McElroy et al. 2014, Bogduk et al. 2013, and Friedman et al. 2006. A maximum score of 20 could be obtained by correctly performing sequential procedural steps within categories of site preparation, anatomy identification using simulated c-arm, needle confirmation, and clean-up. Participants scored 1 point each if correctly demonstrating various graded steps of the procedure (e.g. “cleanse skin with sterile prep’, “spinal needle inserted into skin overlying interlaminar space”, “LOR syringe attached to Touhy needle”).

The primary outcome was the assessed competence as defined by changes in pre and post-skill test scores immediately after intervention and changes in the immediate post-training and 15-week follow-up skill test scores. The secondary outcomes included changes in confidence as defined by changes between pre and post self-graded points immediately after intervention.

Data collection and analysis

All participants were given a subject ID, with only the PI having access to the key. All data were kept on secure servers and all data for analysis was de-identified. Wilcoxon signed-rank test was used within each group to compare changes in pre and post-test scores. Wilcoxon rank-sum test was used between groups with regards to the differences in changes in pre and post-test scores. Data was analyzed using SAS version 9.4 (SAS Institute Inc., Cary, NC).

## Results

**Figure 2:**
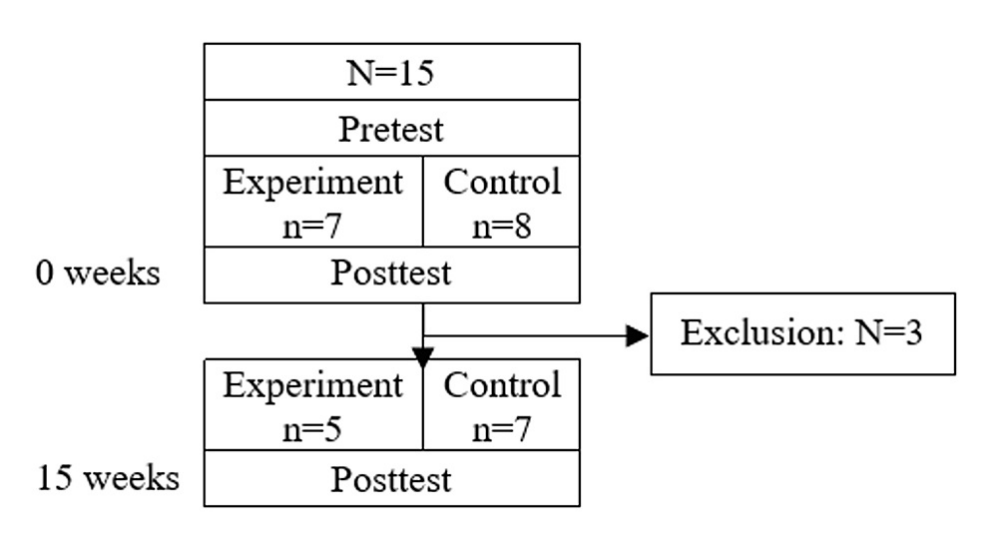
Study Flowchart

A total of 15 PM&R residents from postgraduate year (PGY) 2 to 4 were recruited and consented to participate in the initial study. Seven residents participated in the experiment arm and eight participated in the control arm. Of these, three residents were lost to follow-up at week 15 (2 in the experiment arm, 1 in the control arm) as they were unable to attend the post-test session due to schedule conflicts. Baseline characteristics for the study population and the study flowchart are shown in Table 1 and Figure 2, respectively. There was no statistically significant difference noted with gender, the current level of training, prior pain rotation, and interest in pain medicine when comparing pretest assessed competence scores.

**Table 1:** Trainee Characteristics PGY: Postgraduate year; PM&R: physical medicine and rehabilitation

	Training groups		
	Simulation Group (n, % of group)	Observation Group (n, % of group)	Total (n, % of group)
Subjects	7 (100)	8 (100)	15 (100)
Gender			
Female	2 (28.57)	3 (37.5)	5 (33.3)
Male	5 (71.43)	5 (62.5)	10 (66.7)
Current year of PM&R residency			
PGY-2	3 (42.86)	3 (37.5)	6 (40)
PGY-3	2 (28.57)	5 (62.5)	7 (46.67)
PGY-4	2 (28.57)	0 (0)	2 (13.33)
Prior Experience			
Yes	5 (71.43)	3 (37.5)	8 (53.33)
No	2 (28.57)	5 (62.5)	7 (46.67)
Interest in Pain			
Yes	6 (85.71)	6 (75)	12 (80)
No	1 (14.29)	2 (25)	3 (20)
Handedness			
Right	7 (100)	7 (87.5)	14 (93.33)
Left	0 (0)	1 (12.5)	1 (6.67)

The results of self-perceived competence (confidence) are summarized in Table 2. Level of comfort with lumbar ILESI increased significantly after the intervention in both the hands-on training group (experiment arm) and the observational training group (control arm) (p=0.0004 and p=0.0005 respectively).

**Table 2:** Result of self-perceived competence Scores are evaluated using the Likert scale, with 1 being "Not at all Confident" and 5 being "Completely Confident" Significance tested at p < 0.05 by the t-test

				95% Confidence Interval	
	Pretest Score; Average ± St Dev	Posttest Score; Average ± St Dev	Significance	Lower	Upper
Simulation Group	2.1 ± 0.9	4.0 ± 0.6	0.0004	-2.5	-1.2
Observation Group	1.4 ± 0.7	3.0 ± 0.8	0.0005	-2.2	-1.0

The results of assessed competence immediately following each educational modality are summarized in Table 3 for the observational training group versus the hands-on training group. At week 0, there was a significant increase in post-test scores when compared with the pre-test scores within each group. While the simulation group showed an 11-point improvement in scores (p=0.016), the observation group showed a 7-point improvement in scores (p=0.008). When comparing between the two groups, the improvement in scores was higher in the hands-on training group than the observational training group, and this was marginally significant (p=0.063).

**Table 3:** Results of Assessed Competence at Week 0 Q1 and Q3 represent the 1st and 3rd quartiles. Maximum score possible is 20. *Wilcoxon rank-sum test for comparing two teaching methods. **Obtained from the Wilcoxon signed-rank test.

	Observation Group (n=8)	Simulation Group (n=7)	p-value*
Pretest Score; Median (Q1, Q3)	8 (5, 10)	6 (5,10)	0.560
	Range 3-13	Range 1-11	
Posttest Score; Median (Q1, Q3)	14.5 (13.5, 16.5)	17 (15, 15)	0.062
	Range 12-18	Range 15-20	
Difference in Pre- and Posttest Scores (Q1 difference - Q3 difference)	7 (5.5-9.0)	11 (8,14)	0.063
	Range 3-11	Range 4-14	
p-value**	0.008	0.016	

The comparisons of assessed competence at 15-week follow-up versus immediately following the educational modality are summarized in Table 4. Seven residents completed the follow-up test in the observational training group, while only five residents completed the follow-up test in the hands-on training group. From week 0 to week 15, there was no significant change in test scores in the observational training group (p=0.969) while there was a decrease in test scores in the hands-on training group (p<0.001)

**Table 4:** Comparisons of Assessed competence at Week 0 and Week 15 Q1 and Q3 represent the 1st and 3rd quartiles. Maximum score possible is 20. *Wilcoxon rank-sum test for comparing two teaching methods. **Obtained from the Wilcoxon signed-rank test.

	Observation Group (N=7)	Simulation Group (N=5)	p-value*
Week 0 Score; Median (Q1, Q3)	14.5 (13.5, 16.5)	17 (15,15)	0.062
	Range 12-18	Range 15-20	
Week 15 Score; Median (Q1, Q3)	16 (14, 16)	13 (12,14)	0.082
	Range 13-18	Range 11-16	
Week 15 - Week 0; Median (Q1, Q3)	0 (-1, 2)	-4 (-4,-4)	0.007
	Range -3-(3)	Range -5- (-3)	
p-value**	p=0.969	p < 0.001	

## Discussion

There is increasing interest in the field of pain medicine among PM&R residents. This is a field that requires proficiency in interventional pain procedures. Additionally for PM&R residents, there are ACGME requirements for exposure to interventional pain procedures [1,2,8,9-12]. Despite these factors, the ideal method of teaching relevant procedures during PM&R residency training has not been examined closely. The use of SBME models to facilitate education of procedural skills has been evaluated in other specialties [8,13-19] but its use in educating PM&R residents in interventional pain procedures is not well established. Simulation training facilitates motor skill development without risk of harm to patients, allowing room for errors to be made and corrected, within a low-stress environment [20].

The results of our study demonstrate that both observational education and SBME facilitate the acquisition of ILESI procedural skill acquisition immediately after teaching. When comparing the magnitude of improvement in performance before and immediately after training, the simulation group had greater improvement than the observational group on the same-day teaching, but this was marginally significant (p=0.063). This is not surprising as both observational training and simulator training reinforce motor skill acquisition while emphasizing different aspects of learning. For example, Kopta outlines 3 major stages of motor skill acquisition, namely cognitive, integrative, and autonomous [21]. The cognitive phase involves listening to and watching new procedures performed, reflecting on what the observational group experienced. Prior studies have demonstrated the benefits of only visualization in improving procedural skills [22]. The integrative phase involves hands-on practice and feedback, reflecting what the SBME group experienced [21]. On the other hand, many specialties have demonstrated the benefits of simulation-based training, [8,13-19,23,24] although research on a direct comparison between observational education and SBME is sparse. While our hypothesis was that the simulation group would have a statistically significant improved performance, the lack of statistically significant superiority may be due to several factors. For one, there was a small sample size. Furthermore, the observational group still received education from an experienced clinician performing an ILESI using a high-fidelity model. Not only does observational learning yield skill acquisition benefits [22,25] but the use of a high-fidelity simulator in both groups may have further boosted benefits to the observational group [19,26].

The results of our study suggest that merely learning by doing is not sufficient to retain procedural skills in the long term and that trainees benefit substantially from learning by seeing. At 15 weeks of follow-up, the results of our study suggest observational group had better retention than the simulation group. While we expected the simulation group to demonstrate better retention given the greater magnitude of improvement during the same-day teaching, this was not the case. One potential reason is that in the observation group, the faculty member demonstrated the ILESI on a simulator three times, allowing for the first two steps of Peyton’s teaching approach “demonstration” and “deconstruction” to be repeated. This may result in greater retention compared to undergoing all four steps of Peyton’s teaching approach only once. The lack of repetition in the simulation group may be compounded by the lack of further repetition in performing the procedure. Surgical literature from various specialties on procedural proficiency emphasizes a learning curve requiring a certain frequency of procedure repetition before gaining proficiency [27-29]. Without repetition, a performance decrease may be expected after SBME within months [30]. Another potential reason that may have contributed to these results on follow-up is participant drop-out. In the simulation arm, 5 out of the original seven participants returned. This may have skewed the results if the dropout participants demonstrated strong retention.

This study had several limitations. First, there was a small number of participants in both the observation and interactive arms. This limited the ability to assume normal distribution as well as generalizability. Second, the study participants were recruited from only two academic PM&R residency programs, also limiting generalizability. Third, two separate attendings evaluated the resident participants which may introduce potential observational bias. Fourth, during the follow-up at 15 weeks, residents were not paired to the same attending as in the initial study, potentially confounding evaluations of procedural knowledge. Fifth, at 15-week follow-up, continued education outside the study was not controlled for.

Future studies can recruit a larger number of residents and include multiple institutions to improve statistical power as well as improve generalizability. Additionally, future studies may include a third arm in which residents are taught using both observation and simulation to evaluate for same-day and longer-term retention of procedural skills. A combination of both methods of teaching may result in a more optimal short- and long-term procedural skill acquisition and retention. These further studies would continue to help elucidate the effectiveness of SBME in teaching PM&R residents interventional procedures such as the ILESI.

## Conclusions

Both observational training and SBME training are effective educational modalities in in short-term interventional pain procedural skill training, and although not statistically significant, SBME may be more superior in immediate retention. However, observational training has better long-term retention than hands-on training. Pain Management and Rehabilitation resident training in interventional pain procedures may be optimized by integrating both methods of learning to produce the best short- and long-term results. Future studies with a larger number of participants can help validate the observations made in this study.
